# Significantly increased bone volume in a critical-sized defect model in the rat animal model by transplantation of a stand-alone vascularized periosteal flap

**DOI:** 10.1007/s00068-025-02770-5

**Published:** 2025-02-28

**Authors:** Florian Wichlas, Maximilian Wenzel, Valeska Hofmann, Klemens Trieb, Amelie Deluca, Herbert Tempfer, Andrea Wagner, Andreas Traweger, Sascha Senck, Christian Deininger

**Affiliations:** 1 Department of Orthopedics and Traumatology, Paracelsus Medical University Salzburg, Müllner Hauptstraße 48, Salzburg, 5020 Austria; 2 Department of Traumatology and Reconstructive Surgery, BG Trauma Center Tübingen, Eberhard Karls University, Schnarrenbergstraße 95, 72076 Tübingen, Tübingen, Germany; 3 Institute of Tendon and Bone Regeneration, Spinal Cord Injury & Tissue Regeneration Center Salzburg, Paracelsus Medical University, Strubergasse 22, Salzburg, 5020 Austria; 4 University of Applied Sciences Upper Austria, Roseggerstraße 15, Wels, 4600 Austria

**Keywords:** Bone volume, Critical-sized defect, Rat, Periosteal flap

## Abstract

**Purpose:**

The repair of bony non-unions remains challenging and often requires graft material due to limited availability of autologous bone. The aim of this study was to investigate the potency of a stand-alone pedicled periosteal flap (PF) *versus* a ligated periosteal flap (PFx), an empty defect and a crossover group in terms of newly formed bone in a 5 mm critical-sized defect in the rat femur diaphysis.

**Methods:**

The following 4 treatment groups were formed out of a total of 36 male Sprague Dawley rats: Pedicled periosteal flap, ligated periosteal flap, crossover (each *n* = 10) and empty defect group (*n* = 6). A prospective randomized plate osteosynthesis was performed. The periosteal flap was dissected along with the supplying vessel from the medial femoral condyle with the aid of magnifying glasses and fixed to the plate and to the defect with a suture. Regular radiographic and µ-CT examinations were performed to determine bone volume inside the defect, as well as descriptive histological examinations.

**Results:**

Newly formed bone tissue was measured by Bone Volume / Tissue Volume. The significant highest ratio to the control group was detected in the PF group after 10 weeks (18.77%) compared to the crossover- (11.28%; *p* = 0.0436), the PFx- (10.98%; *p* = 0.0411), and the control group (10.47%; *p* = 0.0293). No relevant differences were found in the descriptive histological examination.

**Conclusion:**

According to the observed results, bony healing of non-union defects can be supported with a pedicled periosteal flap. The superiority of the pedicled compared to the ligated periosteal flap suggests that the improved blood flow within the defect area is an essential component of the healing phase itself.

## Introduction

Successful treatment of bony non-unions is challenging in orthopedic-trauma surgery. Bony nonunions can be observed in high- middle- and low-income countries after high energy trauma and it further seems an overall ubiquitous problem in fracture care [1, 2]. The economy and patient’s financial situation play a significant role in the treatment and outcome of non-unions, affecting everything from access to healthcare and the quality of treatment options to patient compliance and recovery. Addressing these discrepancies requires a multifaceted approach, including policy interventions, insurance reforms, targeted research and developmental efforts to ensure suitable access for an effective treatment. Multiple techniques and concepts have been described, from very basic to sophisticated surgical approaches for the successful treatment of non-unions [3–5]. The use of periosteal flaps (PF) for treating non-unions in bone represent a specific technique to promote fracture healing. This method works by activating the biological tissues surrounding the fracture. The local blood flow to the fracture site is enhanced, which is crucial because increased blood flow delivers essential nutrients and osteogenic elements directly to the site of the non-union. This approach distinguishes itself by directly targeting and improving the conditions needed for bone healing [6–8]. The osteogenic potential of the periosteum is known for its abundant amount of vascular supply in the outer fibrous and its inner cambium layer containing mesenchymal stem cells (MSC) and other factors promoting osteogenesis by intramembranous- and endochondral ossification [6–8]. PF have already been used successfully clinically and in animal models [9–11]. While its clinical application is mostly described in published case series, studies in rodents paradoxically combine the PF with other osteogenic matrices (scaffolds) instead of testing it alone [12, 13]. The question remains how potent the PF is, as a stand-alone technique. This consideration gains importance given that the introduction of additive materials, often inert, can potentially increase the risk of infection. This is particularly pertinent in cases of bony non-unions, where an underlying infection is frequently a contributing factor [14, 15]. As a bony non-union model is validated by creating a critical-size defect (CSD) in a rat femur and PF have shown to be practicable in rats, they seem to be an ideal setting for this study [16].

The aim of this present study was to investigate the bone forming capacity of a stand-alone PF in bony non-unions by CSD models in a rat femur. Further, PFs were tested in a persisting CSD after 5 weeks to determine its osteogenic potential in a persisting non-union. Finally, the potential requirement of a vascular supply of the PF was evaluated by comparing two treatment groups; one with a pedicled periosteal flap and the other one with a ligated one.

## Materials and methods

### Experimental animals

All animal experiments and conducted procedures were in accordance with the law on animal experimentation and are approved by the regulatory authorities of the province of Salzburg, Austria (Permit No. 20901-TVG/134/11-2021 from jan. 19th 2021). The study meets the ARRIVE criteria.

A total of thirty-six Sprague Dawley rats (12 weeks old, weighing approximately 475–550 g; Janvier Labs SAS, France) were used for this study. The animals were randomly assigned to either one of four experimental groups. All animals were kept under standard housing conditions (2–3 rats per cage) with free access to food and water. Post-operatively the same animals were kept in groups of 2 to 3 rats per cage. Rooms were maintained at 25 ± 2 °C and a 12:12 h light/dark cycle, light on at 07:00 h.

### Animal study design

**Fig. 1:**
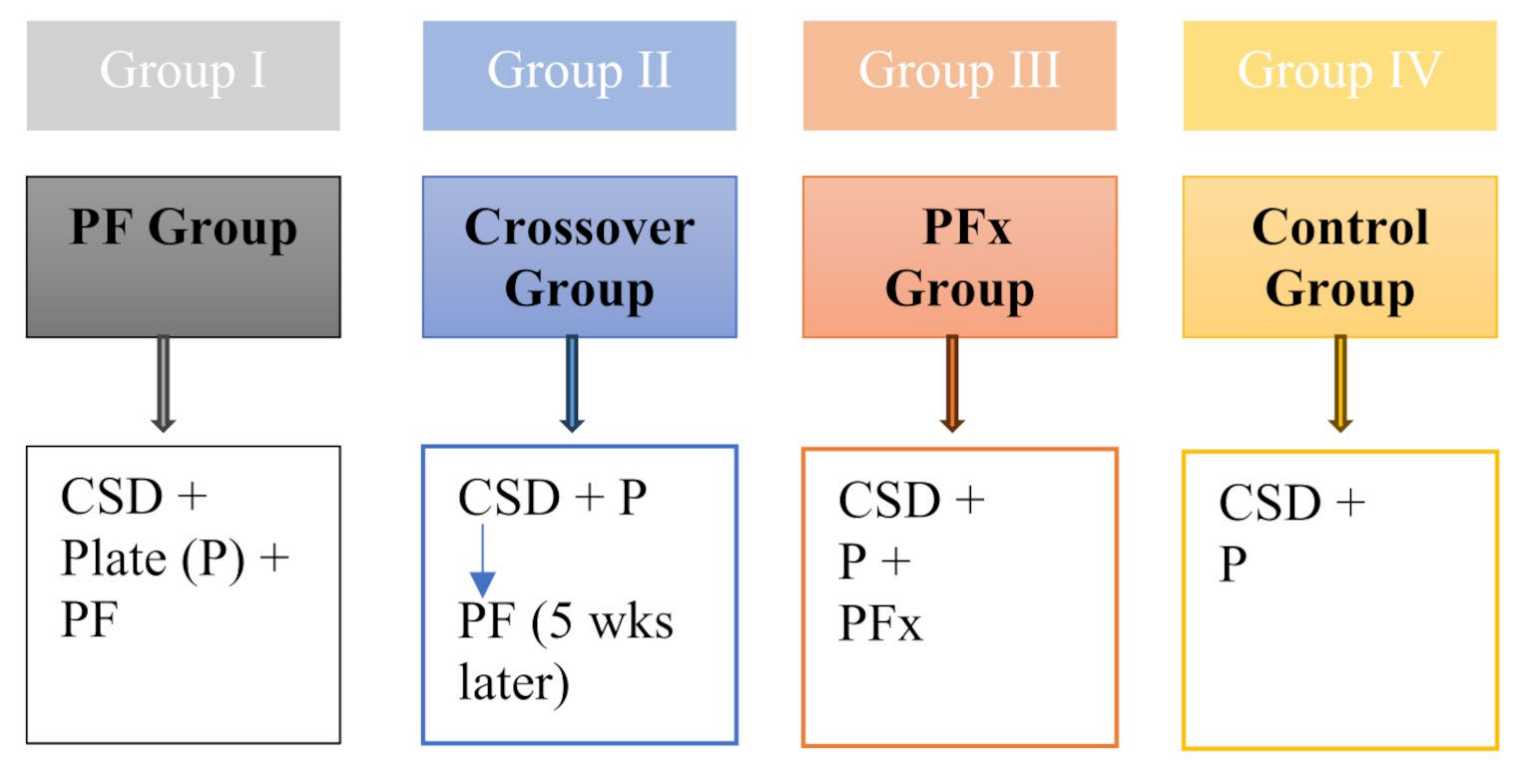
Overview of the study design with 4 animal groups. CSD = Critical Size Defect, P = Plate, PF = Periosteal Flap, PFx = Ligated Periosteal Flap

The study design incorporated 4 groups to which the animals were randomly assigned to as demonstrated in Fig. 1.

Group I – PF group: Incorporated a Critical-size defect (CSD), bridged with a plate (P) and the Periosteal flap (PF) which covered the bony non-union.

Group II - Crossover group: First a CSD was created and stabilized with a plate (P). After 5 weeks, the PF was transplanted through a second surgery.

Group III - Ligated PF (PFx) group: Incorporated a CSD, bridged with a plate (P) and a ligated Periosteal flap (PFx) covered the bony non-union.

Group IV - Control group: Consisted of CSD, which was bridged with a Plate (P).

The aim of the selective group division was that animals in the PF group should induce bony healing within the CSD. The crossover group should allow for evaluation of the efficiency of bony healing in a persistent non-union. The PFx group should assess the necessity of a vascular pedicle and the control group should represent a plate treatment only.

**Fig. 2:**
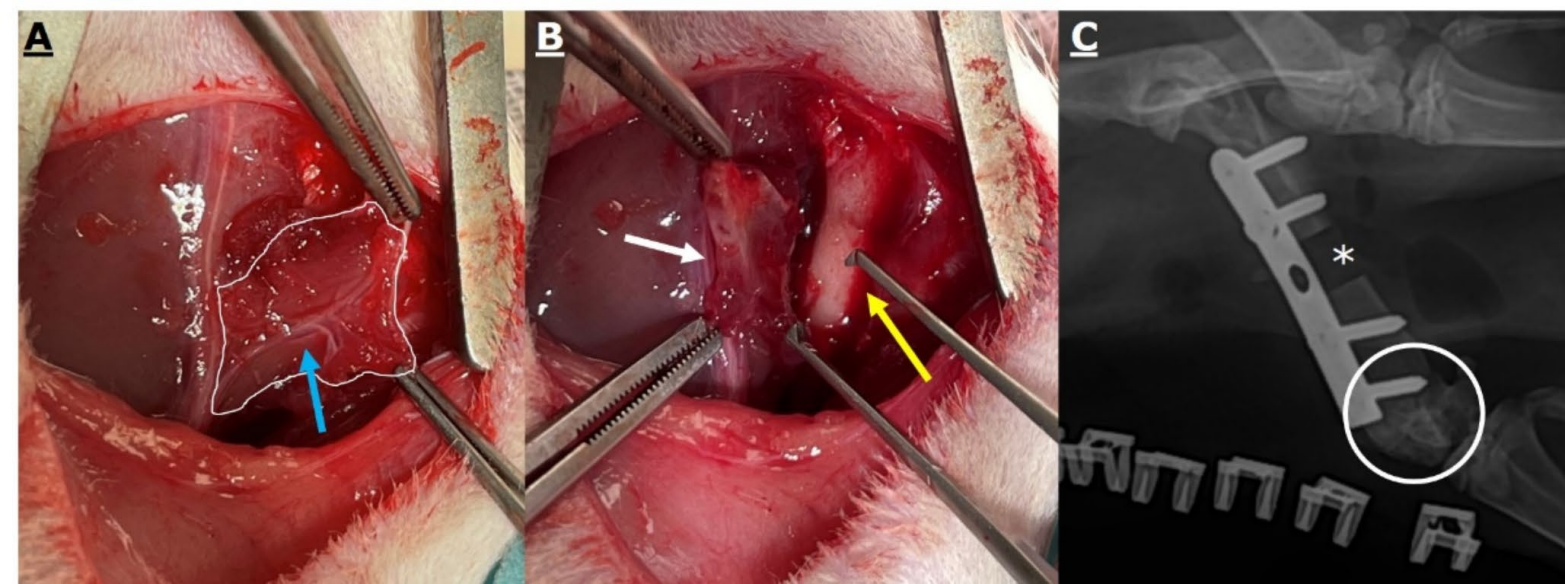
Intraoperative images showing. **A**: periosteal flap (white outline), feeding vessel (blue arrow), and **B**: flipped periosteal flap (white arrow) with exposed medial femoral condyle (yellow arow). **C**: postoperative X-Ray of the plate osteosynthesis applied, the 5.0 mm critical sized defect (*), and the harvesting site (white circle)

### Surgical procedures

After an appropriate acclimatization phase, all rats underwent surgery on the right femur. Thirty minutes preoperatively the rats received 0,03 mg/kg buprenorphine and antibiotics (Clindamycin, 45 mg/kg) subcutaneously (s.c.). Anesthesia was induced in an airtight box with 4% (v/v) isoflurane in oxygen and maintained at 2% (v/v) with a flow rate of 500 ml/min. During surgery, the animals were placed on an electric heating pad to prevent hypothermia (Harvard Apparatus, Holliston, MA, USA). Following shaving and sterile preparation of the surgical area, a skin incision measuring 4–5 cm was made medially along the right femur. A 5-hole angle-stable plate (1.5 mm Aptus titanium locking plate, Medartis, Basel, Switzerland) was attached to the distal femur by drilling 4 holes with a 1.1 mm drill bit (Gebrüder Brasseler, Lemgo, Germany). The central hole was intentionally not secured, leaving it unlocked. A 5 mm CSD was created using a 0.44 mm Gigli wire saw (RISystem AG, Lanquart, Switzerland) distal to the central plate hole. The removed bone fragment was measured using a caliper and the defect site rinsed with sterile saline solution. Depending on the group, a PF was elevated from the medial condyle with a blade after its vascular pedicle was isolated and then rotated into the CSD. The PF was sutured with a single stitch onto the central plate hole. In the PFx group the vascular pedicle was ligated. In the crossover group, the PF was prepared during a second follow-up surgery after 5 weeks. Within the control group, only a CSD was stabilized with a plate (Fig. 2). The wound was shut by sutures in layers. Wound clips were used for closure and removed after 7 days. Magnifying glasses (4x-500, Zeiss, Oberkochen, Germany) were used during the entire operative procedures.

The postoperative analgetic treatment consisted of 0.03 mg/kg buprenorphine s.c. twice a day for three days and 20 mg/kg tramadol-hydrochloride once a day through drinking water for 5 consecutive days. The animals had ad libitum access to food and water and were frequently monitored for complications, weight loss or abnormal behavior. Rats were euthanized (Pentobarbital-Natrium 300 mg/mL; Release; intraperitoneal injection) 10 weeks postoperatively (first surgery) and the femurs harvested for µCT and histological analysis.

### X-Ray control and Microcomputed tomography

X-Ray images, antero-posterior and lateral views, were performed under general anesthesia immediately postoperatively, after 1 and 2 weeks and then in 2-week intervals until the endpoint (10 weeks) by two independent surgeons. The positioning of the rat femur, to obtain replicable images, was constant due to rod fixations of the right leg at the same angle. (X-ray source: Orange 9020HF (EcoRay Co.,Ltd.; Seoul, Korea) Detector: Flat Panel Detector FPD2P(Venu1417P) (iRayTechnology Ltd., Shanghai, China) Software: Conaxx2 VET (PROTEC GmbH & Co. KG, Oberstenfeld, Germany) X-ray source settings: 44 kV, 20mAs).

After 10 weeks, femoral bones and the covering muscles were exarticulated in the hip and knee joint and then placed into 4% paraformaldehyde (PFA) in PBS (phosphate buffered saline). After 24 h, the PFA concentration was reduced to 2% (2 g paraformaldehyde powder (Sarstedt AG and Co, Nümbrecht, Germany) to 100 mL of 1x PBS. µ-CT scans were performed on all samples in this solution.

During the scanning procedure the samples were stored in polymer sample tubes filled with PFA to prevent dehydration, scanning three samples at a time. The samples were scanned at a resolution of 35 μm isometric voxel size using a RayScan 250E cone beam XCT device equipped with a Perkin Elmer flat panel detector (2048 × 2048 pixels with a pixel size 200 μm) and a Viscom 225 kV microfocus X-Ray tube. The X-Ray scanning parameters were set to 180 kV and 200 µA with an integration time of 999 ms. A 0.5 mm physical copper filter-plate was applied to prevent beam hardening artefacts.

Reconstruction of the acquired data was performed using X-AID Software (MITOS GmbH, Garching, Germany) involving a beam hardening correction. Post processing was performed in VGSTUDIOMAX 3.5 (Volume Graphics GmbH, Heidelberg, Germany). Firstly, image data was aligned so that the X-, Y- and Z-axes of the software coordinate system coincide with the frontal, sagittal, and transversal plane respectively. This was done for all samples in order to enable the establishment of a comparable Region of Interest (ROI) throughout the complete datasets. This ROI was chosen to be of cylindrical shape in the center of the diaphysis of the femur. For this purpose, a circle with the diameter of the inner sample tube surface was drawn in the transversal plane and then extruded along the vertical axis. The height of the individual cylinder for each sample was limited by the distance between the two screws nearest to the fracture site. The heads of the two screws were included in the ROI and thus mark the vertical ROI-cylinder boundaries. Subsequently, the region growing tool provided by VGSTUDIOMAX 3.5 was utilized to segment the implant and the screws. This segmentation was then subtracted from the ROI in order to be eliminated from the evaluation. Afterwards, the ROI was extracted and a median filter with a size of 3 × 3 × 3 voxels was applied for the purpose of denoising the images. The region growing tool was then used to segment the bone structure in the ROI. In this tool, a grey value (GV) threshold, which depended on the grey value distribution of the individual measurement, was set with the purpose of excluding liquid areas with GVs similar to bone GVs caused by BH artefacts from the selection. However, this approach was not sufficient to exclude all of the liquid areas. The remaining areas with BH artefacts were subsequently processed manually for each image stack. The finished evaluation contained the bone volume separated from the formalin liquid in the ROI. VGSTUDIOMAX 3.5 provides morphometric data for the evaluation in form of a ratio between Bone Volume and Tissue Volume (BV/TV) in percent, which was chosen as result parameter for the ROI [17–19].

For the samples containing broken implants, an adapted evaluation process was established. After the anatomical alignment of the images, the polyline tool was used to create two three-dimensional ROIs containing the proximal and the distal part of the femur respectively. The proximal femur part was aligned to the distal part using the flat surfaces of the broken implant as reference. After the alignment, the same evaluation process as described above was carried out.

### Histological examination and staining

**Fig. 3:**
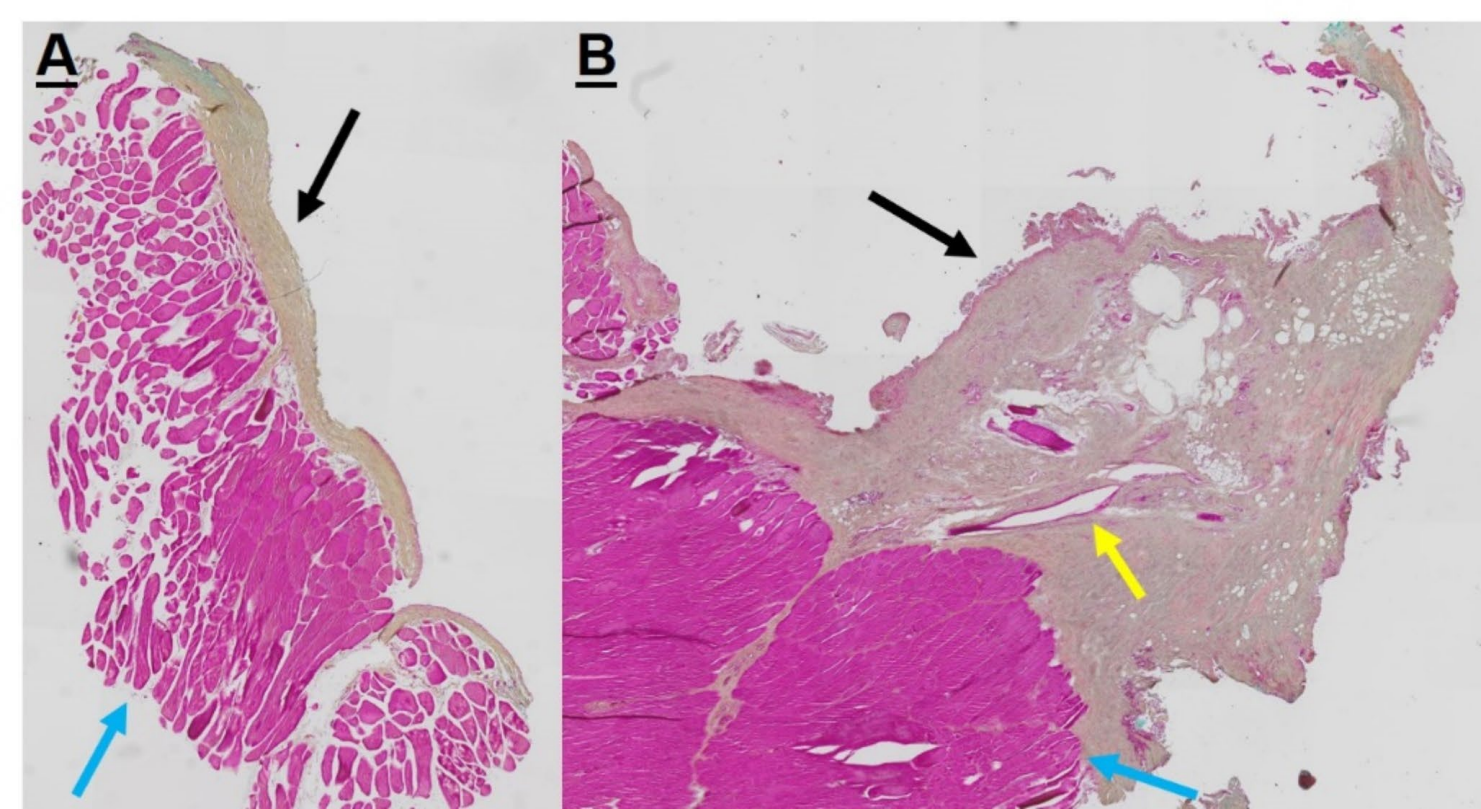
**(A)** Transverse section of a harvested periosteal flap (white arrow) with neighboring musculature (blue arrow). **(B)** Longitudinal section of the periosteal flap with one of the supplying vessels (yellow arrow). Movat Pentachrome staining

After the µ-CT scans, the samples were decalcified in 2% PFA/12.5% EDTA solution (pH = 7.5). After a minimum of 7 weeks the plates were removed and the femora were processed for paraffin embedding and 7 μm sections were deparaffinized using Roti^®^-Histol (Carl Roth, Germany), rehydrated in a graded alcohol series and stained either with Masson-Goldner trichrome, Alcianblue Nuclear Fast Red or Movat’s pentachrome stain (MORPHISTPO, Offenbach, Germany). Digital high-resolution images were acquired with focusing on the CSD area, using a Olympus slide scanner VS 120 (Olympus/Evident, Hamburg Germany). In addition, histological sections of periosteal flaps of the medial femoral condyle of the opposite side were prepared and stained by Movat´s Pentachrome staining (Fig. 3).

### Statistical methods

For comparison of the defect bone volumes determined by µCT analysis (mean ± SD; mm^3^), a One-way ANOVA test with post-hoc pairwise comparisons (Tukey´s) were performed. Samples were tested for normal distribution using the Shapiro Wilk test. For comparison of two groups the unpaired t-test with Welch’s correction was used. Significance was set at α = 0.05. All tests were performed using GraphPad Prism v. 9.02 (La Jolla, CA, USA).

## Results

A total of 43 rats were included in this study. Eleven animals were excluded due to implant failure with plate osteosynthesis fracture observed during routine radiographic examinations (PF Group 2, PFx Group 4, Control Group 4, and Crossover Group 1).

### X-Ray

X-Ray evaluations were carried out to determine plate stabilization and fracture healing progression. Overall, four rats suffered implant failure through a plate breakage within the first 8 weeks postoperatively. Affected animals were euthanized and excluded from the final evaluation.

Observed plate breakages in the last radiographs (10 weeks) were included in the study. They were evenly distributed across the 4 treatment groups and the rats´ behavior and gait pattern were normal. Radiographic evaluation by X-Ray showed no bridging callus in any group.

### BV/TV

**Fig. 4:**
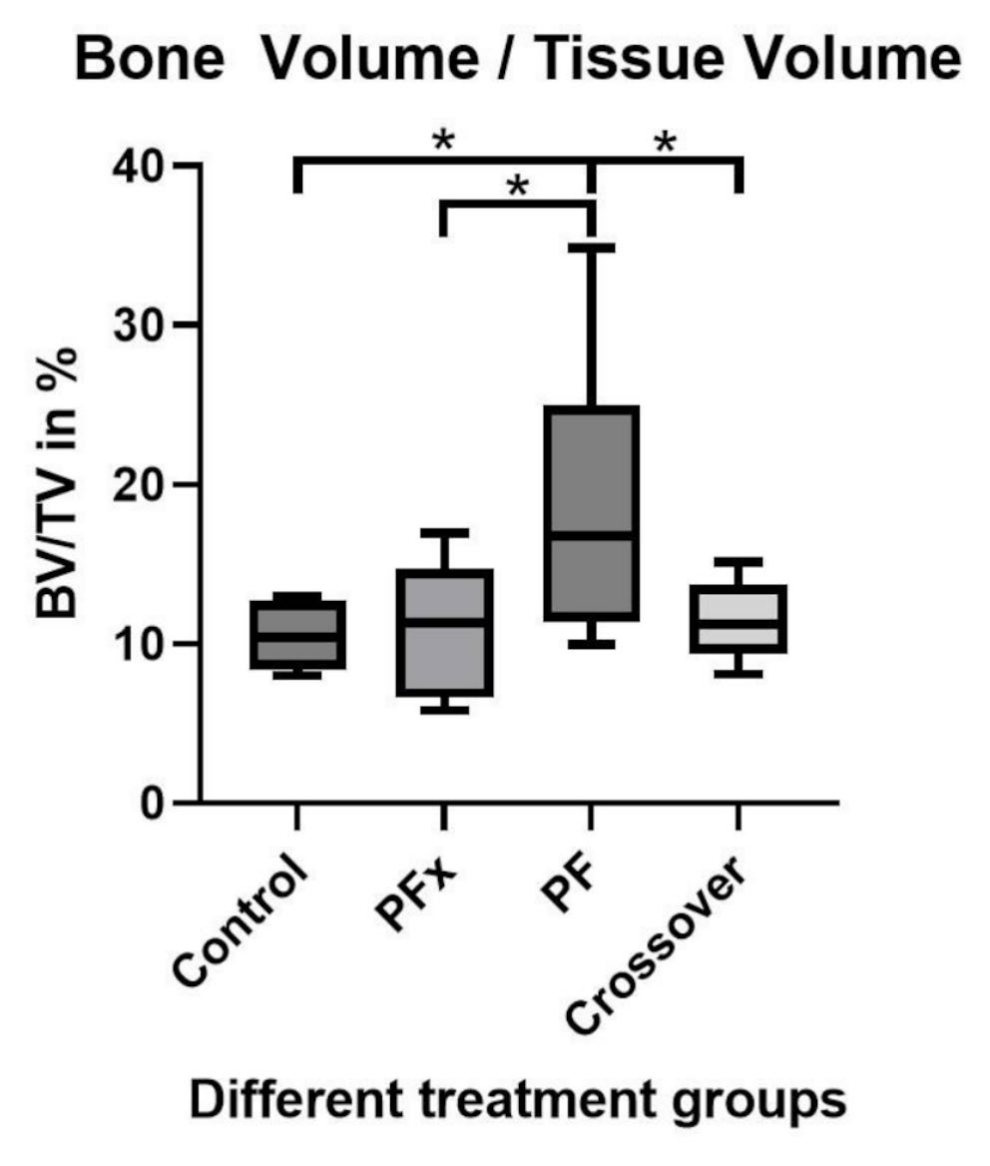
Unpaired t-test with Welch’s correction of the newly formed Bone Volume to Tissue Volume inside the defect. (*p*= *<0.05). The results show a significantly higher BV/TV in the PF group (18.77%) compared to the other 3 groups. Crossover- (11.28%; *p* = 0.0436), the PFx- (10.98%; *p* = 0.0411), and the control group (10.47%; *p* = 0.0293). There were no significant difference between the other groups

Thirty-two rats were evaluated and the results are summarized in Table 1. Newly formed bone tissue was measured by BV/TV. The highest BV/TV ratio was observed in the PF group (18.77%; after 10 weeks, Fig. 4). This was significantly higher than in the crossover- (11.28%; *p* = 0.0436), the PFx- (10,98%; *p* = 0.0411) or the control group (10.47%; *p* = 0.0293).

**Table 1:** Bone Volume/Tissue volume values of the µ-CT imaging evaluation of the different treatment groups

	Control	PFx	PF	Crossover
Number of values	6	9	8	9
Minimum	8.000	5.830	9.980	8.070
25% Percentile	8.375	6.625	11.37	9.395
Median	10.39	11.30	16.77	11.26
75% Percentile	12.69	14.71	24.96	13.67
Maximum	12.94	16.95	34.80	15.13
Range	4.940	11.12	24.82	7.060
Mean	**10.47**	**10.98**	**18.77**	**11.28**
Std. Deviation	2.133	4.073	8.548	2.484
Std. Error of Mean	0.8709	1.358	3.022	0.8281

### Histological analysis

**Fig. 5:**
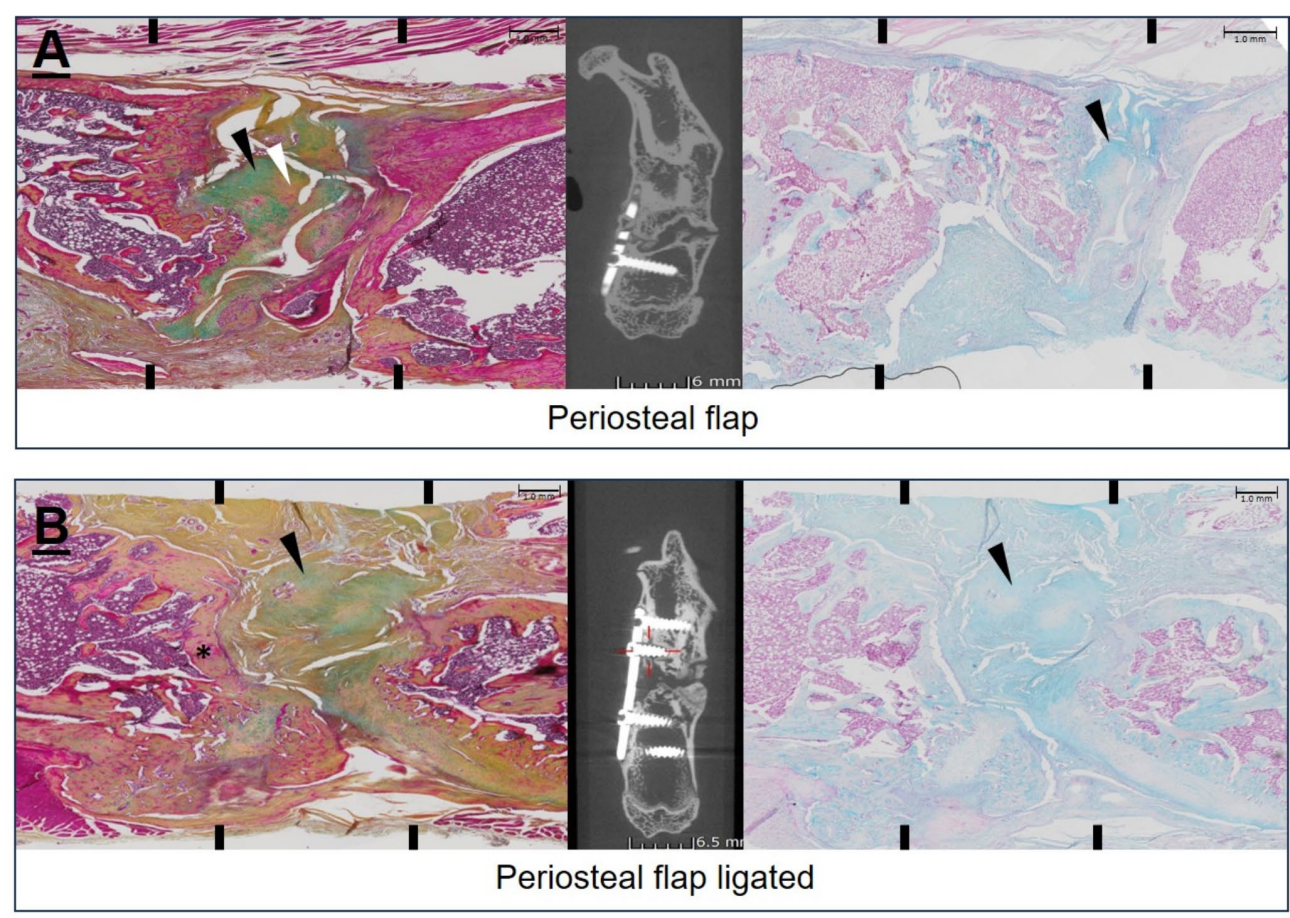
Histological sections of the CSD (Movat Pentachrome (left) and Alcian blue Fast red staining (right)) with corresponding µ-CT images 10 weeks postoperatively. **(A)** Specimen with periosteal flap (PF) shows primary fibrotic tissue (greenish/black arrowhead) and partially calcified areas (yellow/white arrowhead) indicating immature woven bone in the Movat pentachrome staining. In the Alcian blue Fast red staining the GAGs (in blue/black arrowhead) demonstrate the fibrotic tissue. **(B)** Ligated periosteal flap shows “capping” of the defect ends (the medullary cavity has closed with compact bone, asterisk). The central region shows fibrotic tissue (greenish/black arrowhead) also shown in the Alcian blue staining in blue (black arrowhead). **(C)** In the crossover group “capping“ is also present (asterisk) and the defect is filled with fibrotic tissue (greenish/black arrowhead), which is partially calcified but does not correspond to immature woven bone, because there are no visible yellow areas. In the Alcianblue staining a remarkable amount of GAGs (blue/black arrowhead) are present. **(D)** The empty defect shows “capping” (asterisk) and fibrotic tissue (greenish/black arrowhead) in the Movat Pentachrome staining and also visible in blue (black arrowhead) in the Alcian blue staining. Below, the muscle (red/black star) is slightly prolapsed

Figure 5 shows representative histological sections of osteotomy zones of all four treatment groups. Analysed specimens within the PF group showed primary fibrotic tissue inside the CSD with partial calcification. This represents immature woven bone. The specimens of the PFx treated femur showed “capping” at the end of the defected ends. Here the medullary cavity was closed with compact bone. The central region displayed fibrotic tissue. Samples of the crossover group showed “capping” as well. The CSD was filled with fibrotic tissue, which was partially calcified but did not correspond to immature woven bone. Empty defect models presented with “capping” and fibrotic tissue inside the defect zone.

## Discussion

The goal of the study was to evaluate the bone forming potential of periosteal flaps without using any additional matrices.

The bone volume in the PF group was significantly higher than in all other groups. After creation of the defect by osteotomy in the femur, the PF induced osteogenesis and increased bone volume quantitatively in comparison to the other groups. Histologic analysis shows capping in the control, the ligated periosteal flap and the crossover group, however does not reflect the significant advantage of the PF, potentially due to the cutting plane and the irregular structure of the tissue. This osteogenic potential of the PFs was described before, but never as a stand-alone technique [6, 7, 12, 13, 20]. The PF in our study didn’t need a carrier material to enable the bone formation process, which was contrary to other studies. We fixed the PF with a single suture to the plate without any additional fixation after positioning it right onto the CSD. To our knowledge, no other study has shown the potential of bone regeneration by PFs alone without additives. Our results indicated that the bone forming process doesn’t necessarily need a scaffold or other similar structures, rather demonstrated that adequate blood flow to the fracture site as well as hematoma formation are sufficient for new bone formation to occur. It could hence be observed that the novel bone formation through PFs occured by intramembranous- and endochondral ossification [21].

In the crossover group, BV/TV values were higher compared to both the PFx group and the control group (11.28% versus 10.98% and 10.47%, respectively). This marginal, not significant difference may be attributed to the timing of the periosteal flap transplantation, which in the crossover group was performed 5 weeks after the primary surgery. The difference between the PF- and the crossover-group (18.77% versus 11.28%) could be explained by the different time interval, as the time of insertion for the periosteal flap in the crossover-group occurred 5 weeks after the primary surgery. It seems plausible that the periosteal flap, despite its delayed transplantation, contributed to improved bone healing, although significantly less than in the initial PF group. Through a series of X-rays over time it could be observed that the processes of bone formation stopped after 5 weeks and it could not be re-initiated by periosteal flap implantation alone. The reason for this could be explained through the mechanism of healing, which has already ceased. The ligated periosteal flap itself was not able to induce new bone. Hence, bone healing is a process over time and is especially advanced within the first few weeks. It cannot be supported or induced by a stand-alone periosteal flap. Possibly, the formed hemorrhage at the fracture site attracts mesenchymal stem cells of the cambium layer of the periosteal flap by chemotaxis. Contrary to an initial osteotomy, a 5 weeks old osteotomy doesn’t form a hemorrhage anymore and the chemotactic stimulus might be missing.

Further, other osteogenic stimuli might decrease after this time period (5 weeks). Factors like low oxygen, low intensity pulse, and bone morphogenetic protein (BMP) probably be present initially but are not as abundant anymore after 6 weeks [22]. The PF was sutured directly to the plate without any tension to protect the vascular pedicle. It has been shown that tension on the periosteum activates the Wnt- and BMP- signaling pathway to activate the differentiation of osteoblasts [23]. All those theories combined might explain that bony healing has ceased after 6 weeks.

In an additional/future study to enhance the result of our findings, a second osteotomy of the already osteotomized edges could be carried out to induce a new bone forming process. This might support the surgical technique of decorticating the non-union ends before initiating treatment with periosteal flaps. Resecting the bone until it bleeds to observe the “paprika sign”, is commonly accepted in the surgical treatment.

The PFx group showed no significant potential to increase the bone volume. Contrary to the PF group, the blood supply provided by the pedicle was cut. Therefore, the overall blood supply provided by the vascular pedicle seems to be essential for the bone formation process [24]. This necessity of a vascular pedicle is supported by other researchers where the periosteal flap was used with a β-TCP scaffold [14, 20]. Their research also suggests, that only cells of the periosteal flap are not sufficient to increase bone volume. In our study samples of the PFx group showed the least osteogenic potential measured by BV/TV. This is interesting so far, as the induced membrane in the Masquelet technique (which is the use of a temporary cement spacer followed by staged bone grafting), is a recent treatment strategy to manage a posttraumatic bone defect. It provides additional osteogenic cells and factors, although the defect was continually filled with cancellous bone [25]. The expectation of an osteogenic potential between the control- and PF-ground was not met.

The control group demonstrated the expected lack of bony healing as it has been previously published [16].

The research available for periosteal flaps in rodents mostly demonstrated bone forming potential of periosteal flaps in combination with different matrices, such as biodegradable scaffolds, bone marrow derived stem cells, allografts, or bioactive glass. In our study this bone forming potential was solely dependent on the periosteal flap itself. The reason for this was that, in our opinion, it was the most often used technique for bone defects in surgical procedures to stabilize non-unions. In such cases, it is often not advised to add inert carrying materials due to the increased risk of infection, as bone defects mainly result from severe soft tissue trauma such as high graded- closed or open fractures.

The periosteal flap might even be beneficial for the treatment of infection as it increases local blood supply and administers the necessary immune blood cells [8, 26]. The bone forming potential of the vascularized periosteal flap only, suffices to promote a bony consolidation in a CSD in rats, without any accessory adjuncts. In contrast to our approach, most of the published studies focused on bone defect reconstruction using 3 columns: 3D-scaffolds in combination with osteogenic factors and the PF as an enhancer [12, 27, 28]. In these studies, the PF showed an additional benefit to the bone forming capacity of the other factors, scaffolds and cytokines or cells but not as a stand-alone treatment. Vögelin emphasized the advantage of “biological containment and concentration of BMP within the osseous defect” by the PF but no bone formation of the PF with Orthogonal PolyLactic Acid (PLA) and HYaluronic acid (HY) (OPLA-HY) alone in the CSD after 8 weeks [27]. OPLA-HY is a composite material used in bone regeneration research. This material is designed to support the growth and healing of bone tissue. It is often combined with recombinant human bone morphogenetic protein-2 (rhBMP-2) and used with a vascularized periosteal flap to enhance bone formation in critical-sized defects, such as those in the femur of rats. More recent studies combined the PF with MSCs (Mesenchymal Stem Cells) and EPCs (Endothelial Progenitor Cells) and found similar enhancing capabilities of the PF, However, the latter studies corroborate our findings that the PF has bone forming capabilities alone with a scaffold only and without the other factors like MSC and EPC [10, 20].

### Article focus

- Newly formed bone in a 5 mm critical-sized defect in the rat femur diaphysis.
- Pedicled periosteal flap have the highest bone forming capacity.

### Key messages

- Newly formed bone tissue measured by BV/TV was the highest in the PF group.
- PFs to form new bone do not need a scaffold or other similar carrying structures.

### Strengths and limitations

The present study had several limitations. First, the animals were euthanized 10 weeks post-femoral surgery, and therefore it was not possible to determine the long-term outcome of the treatments on bone remodeling. The underlying cellular and molecular mechanisms driving the synergistic effects of bone formation with a PF remain unclear.

## Data Availability

No datasets were generated or analysed during the current study.
